# In Search of Variables Affecting Mental Adjustment and Acceptance of Cancer among Urological Patients

**DOI:** 10.3390/jcm13133880

**Published:** 2024-07-01

**Authors:** Adrianna Królikowska, Marzanna Stanisławska, Małgorzata Starczewska, Anita Rybicka, Kamila Rachubińska

**Affiliations:** Department of Nursing, Pomeranian Medical University in Szczecin, Żołnierska 48, 71-210 Szczecin, Poland; adrianna.krolikowska@pum.edu.pl (A.K.); marzanna.stanislawska@pum.edu.pl (M.S.); malgorzata.starczewska@pum.edu.pl (M.S.);

**Keywords:** cancer, acceptance of cancer, mental adjustment to cancer

## Abstract

**Background/Objectives:** Genitourinary cancers are now considered a major problem in modern medicine. In urological oncology, the most frequently occurring diseases are prostate, bladder and renal cancer. Any cancer has a profound effect on the life of a patient. Therefore, disease acceptance and mental adjustment to the condition are the key elements in coping with cancer. Aim: The main aim of the study was the determination of the level of acceptance of illness and mental adjustment to cancer in urological patients undergoing surgical treatment and the assessment of the effect of mental adjustment on disease acceptance. **Material and Methods:** The study group comprised 150 patients treated at the Department of Urology and Urological Oncology at the Independent Public Clinical Hospital No 2 in Szczecin. The study made use of the diagnostic survey method with the original questionnaire and standardized research tools: Acceptance of Illness Scale (AIS) and Mental Adjustment to Cancer Scale (Mini-MAC). **Results:** The analysis of mental adjustment to cancer according to Mini-MAC revealed that the respondents most frequently adopted the fighting spirit strategy (M; 22.22). Slightly less frequently adopted strategies were positive re-evaluation (M; 21.28) and anxious preoccupation (M; 17.07). The least frequently adopted strategy was the helplessness-hopelessness strategy (M; 13.14). The analysis of data showed a statistically significant negative correlation (r = −0.245; *p* = 0.003) between disease acceptance according to AIS and age. The data analysis revealed a statistically significant positive correlation with helplessness-hopelessness and destructive style (*p* < 0.05). **Conclusions:** Mental adjustment to cancer was found to affect the acceptance of illness. When providing comprehensive care to cancer patients, it is equally crucial to consider the physical as well as mental health aspect, taking into account the aforementioned factors which affect both acceptance as well as adjustment to disease.

## 1. Introduction

Genitourinary cancers are currently regarded as a major issue in modern medicine. In urological oncology, the most frequently occurring diseases include prostate cancer, bladder cancer and renal cancer [1]. There are numerous factors that increase the risk of cancer [2,3]. Both genetic predispositions as well as environmental factors have a fundamental role in the development of cancer [4]. Treatment of cancer rests on taking actions aimed at providing the maximum effectiveness of treatment [5]. Currently, various methods of therapeutic procedures are available in oncology. Local treatment options, i.e., surgical treatment or radiotherapy, as well as systemic treatment options such as chemotherapy or hormonal therapy, are generally applied [6,7,8,9]. Also, particular importance lies in cancer prophylaxis, understood as overall activities aimed at preventing cancer through early diagnosis and treatment [10].

Any cancer has a profound effect on a patient’s life. The diagnosis initially triggers numerous negative emotions, a sense of threat, stress, fear and the feeling of uncertainly. It creates heavy psychological burden and imposes certain limitations and changes to the lives of the patients and those closest to them [11,12]. Additionally, the limitations due to cancer may translate into various social dysfunctions [13]. Nevertheless, in the course of the disease, it is possible to achieve a level of adaptation to the disease, followed by disease acceptance, which is directly related to adjustment to cancer. Other variables affecting the quality of life and acceptance of the disease that could improve the quality of tests are not taken into account. Urinary diversion plays a key role in this case. In a broad perspective, this is understood as effective coping with a traumatic event, including both the diagnosis of the disease as well as its consequences [14]. Acceptance and adjustment to disease occur by means of adopting a certain strategy of coping with cancer. The following styles of fighting the disease are distinguished: the destructive style and the constructive style. The destructive or passive style consists in perceiving the disease as a threat, a disturbing and uncontrollable event which results in decreased mood, the occurrence of anxiety disorders and the feelings of helplessness-hopelessness, being lost and alone and confusion, which consequently leads to passive submission to illness. The constructive or active style is understood as actively fighting the disease, which is manifested by the active pursuit of regaining full physical function and hope for recovery [15,16,17].

Adjustment to cancer, mainly due to adopting constructive strategies of fighting the disease and acceptance of illness, is the key element in coping with cancer. An optimistic attitude provoking perception of the disease as a challenge and encouraging the patient to fight the disease actively, as well as the ability to cope with said situation in a way which, while recognising the gravity of the situation, allows hope are crucial for achieving better therapeutic effects [18]. Patients who are capable of accepting the disease are much more willing to fight for recovery. Acceptance of cancer is considered the major indicator of adjustment to a traumatic event [19]. Higher level of disease acceptance translates into improved adjustment and higher quality of life. Additionally, it has a direct effect on improved functioning in the physical, mental, social and spiritual spheres, thus creating interaction between the requirements due to oncological disease and the resulting treatment with the individual ability to react and respond to burden due to cancer [20,21].

The aim of the present study was the search for variables affecting the acceptance and mental adjustment to cancer in patients undergoing surgical urological treatment.

## 2. Materials and Methods

The study group comprised 150 patients diagnosed with genitourinary cancer treated at the Department of Urology and Urological Oncology at the Independent Public Clinical Hospital No 2 in Szczecin. Prior to conducting the research, the approval of the Bioethical Committee of Pomeranian Medical University in Szczecin (Resolution No. KB-0012/46/01/2013) and the approval of the hospital management were obtained. The study was conducted in accordance with the Declaration of Helsinki. Participation in the study was voluntary and anonymous, and the respondents were informed about their right to withdraw consent to participate at any time. The proper course of the study was substantively supervised. The inclusion criteria were a confirmed diagnosis of genitourinary cancer treated by means of surgical methods and informed consent of the participants. The study was based on the diagnostic survey method using the questionnaire survey technique:

The original questionnaire and the following standardized research tools:

Acceptance of Illness Scale (AIS)—used to assess the patient’s level of coming to terms with the disease. It consists of eight statements describing negative consequences of poor heath due to a particular diagnosis. The answers are given on a five-grade scale with a score range of 8–40 points. With respect to said scale, there are no specific standards determining the score which would indicate a high or low level of disease acceptance. However, it is assumed that higher scores represent higher acceptance levels, whereas lower scores indicate lower levels of disease acceptance [22,23]. Cronbach’s coefficient is α = 0.82, and construct validity explains 49% of the total variance [24].

Mental Adjustment to Cancer (Mini-MAC)—designed to assess cancer coping strategies, consisting of 29 items describing responses of patients. Each item is scored on a four-grade scale. The tool makes it possible to determine the frequency of adopting a particular style of coping with cancer and refers to the strategies of positive re-evaluation, fighting spirit, the concept of anxious preoccupation and the helplessness-hopelessness strategy. The score for each strategy is 7–28 points. It is assumed that the higher the score is, the greater the frequency of behaviour associated with the particular coping style is. Additionally, Mini-MAC allows for the assessment of the following two styles of coping with cancer, both of which are comprised of two strategies:

- the constructive coping style (fighting spirit strategy and positive re-evaluation strategy), and
- the destructive style (strategy of anxious preoccupation and helplessness-hopelessness strategy).

The results for both styles fall within 14–56 points. Higher scores represent a higher frequency of behaviour associated with a particular coping style. The sten standards of each style allow interpretation of the score. It is generally acknowledged that sten scores 1–4 are to be interpreted as low, 5 and 6 as average and 7–10 as high [25,26]. Cronbach’s alpha coefficient for said scale is 0.87–0.92 for particular strategies [27].

### Statistical Analysis

The analysis of the quantitative variables, i.e., expressed numerically, was conducted by means of calculating the mean, standard deviation, median and quartiles. The analysis of the qualitative variables, i.e., expressed non-numerically, was conducted by means of calculating the number and the percentage of occurrences of each response. The correlations between the quantitative variables were analysed with the use of Spearman’s correlation coefficient. The adopted significance level was 0.05. Consequently, *p* values less than 0.05 were interpreted as indicating significant relationship. The analysis was performed using R software, ver. 4.1.2 [28].

## 3. Results

The study group comprised 150 patients. The vast majority of the surveyed were men (70.67%). The mean age was 62. The majority of respondents had vocational education (32.00%), were residents of a city between 10,000 and 100,000 in population (28.67%) and were in a formal relationship (63.33%). Among the study group, there was a predominance of patients with a diagnosis of bladder cancer (37.33%), followed by prostate cancer (32.00%) and renal cancer (29.33%). All patients had undergone a surgical treatment (100%) (Table S1).

The mean score according to AIS representing the acceptance of disease amounted to 29.68; therefore, the respondents were found to manifest the neutral attitude towards cancer, leaning towards acceptance.

The analysis of mental adjustment to cancer according to Mini-MAC revealed that the respondents most frequently adopted the fighting spirit strategy (M; 22.22). Slightly less frequently adopted strategies were positive re-evaluation (M; 21.28) and anxious preoccupation (M; 17.07). The least frequently adopted strategy was the helplessness-hopelessness strategy (M; 13.14) (Table 1).

The data analysis showed that more than half of the participants (53.33%) manifested an average level of the constructive style, and 54.67% manifested a low level of the destructive coping style (Table 2).

The analysis of data showed a statistically significant negative correlation (r = −0.245; *p* = 0.003) between disease acceptance according to AIS and age.

However, there were no statistically significant differences (*p* > 0.05) in terms of acceptance of illness depending on the sex of the respondents, marital status, location of the cancer, time since the diagnosis or family history of cancer.

The analysis of own research revealed statistically significant differences (*p* < 0.05) with respect to disease acceptance according to AIS depending on the patients’ education, place of residence, frequency of hospitalization, having undergone chemotherapy, surgical treatment and the type of the applied procedure. The level of disease acceptance was markedly higher among patients with vocational and secondary education (*p* < 0.001), residents of medium-sized and large cities (*p* = 0.01) and among patients hospitalized once, as well as patients not treated with chemotherapy (*p* = 0.027). Among the group of respondents who had traditional surgery (*p* = 0.002) and those after cystectomy (*p* < 0.001), the level of disease acceptance was determined as markedly lower in comparison with other groups (Table S2).

The data analysis revealed a statistically significant positive correlation with helplessness-hopelessness (r = 0.242, *p* = 0.003) and the destructive style (r = 0.197, *p* = 0.016). There were no statistically significant differences (*p* > 0.05) in mental adjustment to cancer according to Mini-MAC among the respondents depending on their sex, place of residence or location of the cancer (Table 3).

Data analysis showed statistically significant differences (*p* < 0.05) regarding the mental adjustment to cancer according to Mini-MAC depending on education. Anxious preoccupation was more severe in patients with primary education than in other groups. Additionally, helplessness-hopelessness and the destructive style were found to be markedly more severe in patients with primary education than in other groups; the results also show greater severity among respondents with vocational education as compared with respondents with higher education. There were statistically significant differences (*p* < 0.05) regarding mental adjustment to cancer according to Mini-MAC depending on the marital status. Anxious preoccupation and the destructive style were more severe among singles in comparison with respondents from other groups. Also, helplessness-hopelessness was found to be significantly more severe among singles than in respondents in a formal relationship (Table S3). 

Data analysis showed a statistically significant positive correlation with helplessness-hopelessness and the destructive style (Table 4).

The conducted analysis of data demonstrated statistically significant differences (*p* < 0.05) in terms of mental adjustment to cancer depending on the frequency of hospitalization, type of treatment and type of procedure. Positive strategies of fighting cancer, i.e., fighting spirit, positive re-evaluation and the constructive style, were found to be predominant in the group of patients not treated with chemotherapy. However, the constructive style was significantly less intense among patients after cystectomy in comparison with other groups. Additionally, helplessness-hopelessness and the destructive style were more severe in patients with a history of several hospitalizations and in the group of patients after chemotherapy as well as in the group of patients after cystectomy. Anxious preoccupation was more severe in the group of patients after bladder removal surgery, and in patients after prostatectomy, it was markedly more intense than among patients after TURB procedure (Table S4). 

The influence of mental adjustment to cancer according to Mini-MAC on disease acceptance according to AIS is as follows.

The results of our own studies revealed a statistically significant positive correlation between fighting spirit (*p* = 0.002), as well as the constructive style according to Mini-MAC (*p* = 0.002) and disease acceptance according to AIS. The analysis also demonstrated a statistically significant negative correlation between anxious preoccupation (*p* < 0.001), helplessness-hopelessness (*p* < 0.001) and the destructive style according to Mini-MAC and disease acceptance according to AIS (*p* < 0.001) (Table 5).

## 4. Discussion

The results obtained in the course of own studies using the Acceptance of Illness Scale (AIS), allowing the determination of the level of coming to terms with the disease, show that the respondents manifested a neutral attitude, leaning towards disease acceptance. It was found that acceptance of cancer was markedly higher in younger patients, respondents with vocational and secondary education and residents of medium-sized and large cities. Furthermore, disease acceptance was found to be significantly lower among patients with a history of several hospitalizations, after cystectomy and those who underwent traditional surgery or chemotherapy [16].

As transpires from the results of our own studies, the vast majority of respondents were men, with the predominance of the age group of 61–70. Most respondents had vocational education, were residents of cities of 10,000–100,000 in population and in a formal relationship. The research conducted by Pieniążek M. et al. on disease acceptance among patients with genitourinary cancers and the study by Zielińska-Więczkowska H. et al. on the acceptance of cancer and its influence on quality of life among the elderly under inpatient and in-home palliative treatment also showed the predominance of men, aged 60–70, with vocational education, residents of cities and in a formal relationship. Similarly, the study by Pieniążek M. et al. showed that the most numerous group of respondents were men, most of whom were 60–70 years old, with secondary and vocational education, residents of cities and claiming to be in a formal relationship [17]. Also, the study by Zielińska-Więczkowska H. et al. showed that the vast majority of respondents were men; in the early old age, i.e., 60–74; with vocational education; residents of large cities and in a formal relationship [29]. The study by Malinowska K et al. indicated that men demonstrate much higher level of disease acceptance than women [30]. However, as was found by Catoo J. et al. in their study on the quality of life among bladder cancer patients, age may be of the greatest importance in determining the quality of life [31].

Acceptance of illness is a key factor in coping with numerous diseases, particularly cancer [32]. The results of the present study conducted with the use of disease acceptance (AIS) show that the respondents manifested a neutral attitude, leaning towards disease acceptance [33]. Similarly, the study by Wojtas K. et al. showed a comparable general score of acceptance of illness, which also indicates an average level of disease acceptance bordering with a high level of acceptance of illness [34]. Moreover, the studies by Piotrowska R. et al. and by Braniecka-Woźniak et al. showed the general level of acceptance of cancer as average [35,36]. Yet another study by Smoleń E et al. concerning the determinants of acceptance of illness among cancer patients presented the average level of disease acceptance [37]. In their study on the level of acceptance of illness and quality of life among lymphoma patients, Ślusarska B et al. demonstrated that the level of disease acceptance in the study group was also average, yet the score according to AIS was found to be lower [38]. However, unlike the results of the present study showing the relationship between the level of disease acceptance and sociodemographic and medical variables, the study by Wojtas K. et al. on the acceptance of cancer among young adults did not identify correlations with demographic variables and variables related to the course of the disease [38]. Nevertheless, the results of our own study demonstrate much higher disease acceptance among younger patients, those with vocational and secondary education and residents of medium-sized and large cities. It was also found that acceptance of illness was markedly lower among patients with a history of several hospitalizations, after cystectomy, traditional surgery and chemotherapy. Partially similar results were presented by Kołpa et al. in their study on determinants of the acceptance and adjustment to cancer, which confirmed the relationship between age and the level of disease acceptance [39]. Another study showing comparable results was conducted by Kupcewicz et al. regarding the effect of the selected sociodemographic variables on the level of acceptance of illness and quality of life; it was found that sex, education and frequency of hospitalization had a statistically significant effect on the adjustment to oncological disease [40].

With respect to mental adjustment to cancer, the data obtained using Mini-MAC, both in the course of the present study as well as presented in other studies, show differences in intensity of attitudes and strategies of coping with oncological disease [41]. The analysis of our own results concerning mental adjustment to cancer according to Mini-MAC demonstrated that the most frequently adopted strategies were fighting spirit and positive re-evaluation, while slightly less frequently adopted strategies were anxious preoccupation and helplessness-hopelessness. It was estimated that more than half of oncological patients manifested an average level of the constructive style, slightly fewer patients manifested a high level of the constructive style and the minimum patient group manifested a low level of the destructive style. Similar conclusions were presented in the study by Glińska J. et al. on the mental adjustment of oncological patients after surgical treatment. The most preferred strategies of coping with the disease were fighting spirit and positive re-evaluation. By far, fewer patients adopted anxious preoccupation and helplessness-hopelessness strategy. The analysis also demonstrates that the constructive style was predominant among most respondents, and the destructive style remained low [42]. Similar results were obtained by Rogala D. et al. who identified fighting spirit and positive re-evaluation strategies to be predominant, whereas anxious preoccupation and helplessness-hopelessness was found to have below-average values. Therefore, the results of our own studies, as well as those presented by Glińska J et al. and Rogala D. et al., demonstrate that the constructive style of coping with cancer was predominant, and the destructive strategies were adopted, by far, less frequently. Furthermore, according to the study by Wiedler-Huszla S. et al. on patients diagnosed with gynaecological cancer, the constructive style of fighting the disease was prevalent, and AIS scores were found to positively correlate with the intensity of the constructive style of mental adjustment and negatively correlate with the intensity of the destructive style [43,44]. Likewise, the study on patients diagnosed with colorectal cancer identified a positive correlation between quality of life and fighting spirit as well as positive re-evaluation strategies and negative correlations with helplessness-hopelessness and anxious preoccupation [45]. However, different results were obtained by Humeniuk E. et al., who identified the highest values of adjustment with respect to anxious preoccupation and positive re-evaluation and the lowest values with respect to fighting spirit and helplessness-hopelessness strategies. Additionally, contrary to results of the present study, the respondents scored markedly higher in the destructive style rather than the constructive style of coping with the disease. Different strategies of coping with oncological disease adopted by the patients may produce disparate results [46]. In a study on psychological resilience in patients with bladder cancer after radical cystectomy, the mean resilience score was 53.45 ± 6.22. With this type of surgery, the most difficult issue is the urinary diversion, rather than the operation and the cancer itself. In patients with an ileal reservoir for continent urinary diversion, disease acceptance and final functional outcomes are at a very high level. It was concluded that more attention should be paid to the psychological state of patients when it comes to treatment. A good attitude towards the disease, a positive coping style and hope for recovery increase patients’ resilience and improve their lives [47,48]. Scientific research proves that mental adjustment has the primary influence on acceptance of illness among oncological patients [49].

An important limitation of the study was that other variables affecting the quality of life and acceptance of the disease that could improve the quality of tests were not taken into account. The urinary diversion, in this case, plays a pivotal role.

## 5. Conclusions

Mental adjustment to cancer identified among the patients under study was found to affect the acceptance of illness. The level of acceptance was higher when associated with the increased intensity of fighting spirit and positive re-evaluation strategies, and lower when it coincided with the increased intensity of anxious preoccupation, helplessness-hopelessness and the destructive style.

There is a noteworthy relationship between age, education, place of residence, frequency of hospitalization, having undergone chemotherapy, surgery method and the type of the procedure with the acceptance of oncological disease, as well as between age, education, marital status and mental adjustment to oncological disease. The study also identified the relationship between the form of the applied treatment and the acceptance and adjustment to oncological disease. When providing comprehensive care to cancer patients, it is equally crucial to consider the physical as well as mental health aspect, taking into account the aforementioned factors which affect both acceptance as well as adjustment to disease.

## Figures and Tables

**Table 1:** Disease acceptance according to AIS and strategies of mental adjustment to cancer according to Mini-MAC (N = 150).

AIS and Mini-MAC		M	SD	Me	Min–Max	Q1–Q3
AIS		29.68	8.53	32	8–40	24–37
Mini-MAC	anxious preoccupation	17.07	4.42	17	7–27	14–20
	fighting spirit	22.22	3.14	22	12–28	21–24
	helplessness-hopelessness	13.14	4.11	13	7–23	10–15
	positive re-evaluation	21.28	3.04	21	13–28	19–24

M—mean, SD—standard deviation, Me—median, Min.—minimum value, Max.—maximum value, Q1—lower quartile, Q3—upper quartile.

**Table 2:** Levels of mental adjustment to cancer according to Mini-MAC.

Styles of Mental Adjustment to Cancer according to Mini-MAC	Interpretation	n	%
constructive style-score	low level	5	3.33
	average level	80	53.33
	high level	65	43.33
destructive style-score	low level	82	54.67
	average level	57	38.00
	high level	11	7.33

n—the number of respondents, %—percentage.

**Table 3:** Mental adjustment to cancer according to Mini-MAC depending on age.

Mini-MAC	Age
	Spearman’s Correlation Coefficient
anxious preoccupation	r = 0.135, *p* = 0.099
fighting spirit	r = −0.11, *p* = 0.179
helplessness-hopelessness	r = 0.242, *p* = 0.003
positive re-evaluation	r = 0.13, *p* = 0.111
constructive style	r = 0.011, *p* = 0.892
destructive style	r = 0.197, *p* = 0.016

r—Spearman’s correlation coefficient.

**Table 4:** Mental adjustment to cancer according to Mini-MAC depending on the time since the diagnosis of cancer.

Mini-MAC	Time Since the Diagnosis of Cancer
	Spearman’s Correlation Coefficient
anxious preoccupation	r = 0.125, *p* = 0.127
fighting spirit	r = −0.123, *p* = 0.132
helplessness-hopelessness	r = 0.284, *p* < 0.001
positive re-evaluation	r = −0.107, *p* = 0.194
constructive style	r = −0.132, *p* = 0.107
destructive style	r = 0.21, *p* = 0.01

r—Spearman’s correlation coefficient.

**Table 5:** The effect of mental adjustment to cancer according to Min-MAC on disease acceptance according to AIS.

Studied Characteristics	Spearman’s Correlation Coefficient	*p*
anxious preoccupation and AIS	−0.395	*p* < 0.001
fighting spirit and AIS	0.251	*p* = 0.002
helplessness-hopelessness and AIS	−0.477	*p* < 0.001
positive re-evaluation and AIS	0.186	*p* = 0.023
constructive style and AIS	0.251	*p* = 0.002
destructive style and AIS	−0.475	*p* < 0.001

*p*—statistical significance coefficient.

## Data Availability

The data presented in this study are available upon request from the first author.

## Supplementary Materials

The following supporting information can be downloaded at: https://www.mdpi.com/article/10.3390/jcm13133880/s1, Table S1. The characteristics of sociodemographic and medical variabilities of the study group; Table S2. Disease acceptance according to AIS depending on sociodemographic and medical variables; Table S3. Mental adjustment to cancer according to Mini-MAC–sociodemographic data; Table S4. Mental adjustment to cancer according to Mini-MAC–medical data.
