# Supplementary material for: In Search of Variables Affecting Mental Adjustment and Acceptance of Cancer among Urological Patients
Source: J Clin Med. 2024 Jul 1;13(13):3880. doi: 10.3390/jcm13133880 (PMC11242229; doi:10.3390/jcm13133880)
Supplement: Supplementary file 1 [file jcm-13-03880-s001.zip › jcm-3025836-supplementary.pdf]

Table S1. The characteristics of sociodemographic and medical variabilities of the study group.

	n	%
**Sex**		
Women	44	29.33
Men	106	70.67
**Age**		
30-40	5	3.33
41-50	17	11.33
51-60	41	27.33
61-70	55	36.67
71-80	27	18.00
81-90	5	3.33
**Education**		
primary	24	16.00
vocational	48	32.00
secondary	47	31.33
higher	31	20.67
**Place of residence**		
Village	38	25.33
city up to 10,000 residents	38	25.33
city 10,000-100,000 residents	43	28.67
city of more than 100,000 residents	31	20.67
**Marital status**		
in a formal relationship	95	63.33
in an informal relationship	19	12.67
single	36	24.00
**Location of the cancer**		
prostate gland	48	32.00
urinary bladder	56	37.33
kidneys	44	29.33
other	0	0.00
prostate, bladder and kidneys	1	0.67
prostate and other location	1	0.67
**Form of treatment**		
chemotherapy	20	13.33
radiotherapy	3	2.00
hormonal therapy	1	0.67
surgical procedure	150	100.00

n – the number of respondents, % - percentage

Table S2. Disease acceptance according to AIS depending on sociodemographic and medical variables.

**AIS and Mini-MAC**		**M**	**SD**	**Me**	**Min-Max**	**Q1-Q3**
AIS		29.68	8.53	32	8-40	24-37
	anxious preoccupation	17.07	4.42	17	7-27	14-20

Mini-MAC	fighting spirit	22.22	3.14	22	12-28	21-24
	helplessness-hopelessness	13.14	4.11	13	7-23	10-15
	positive re-evaluation	21.28	3.04	21	13-28	19-24

M- mean, SD- standard deviation, Me – median, Min.- minimum value, Max.- maximum value, Q1-lower quartile, Q2-upper quartile

Table S3. Mental adjustment to cancer according to Mini-MAC–sociodemographic data .

Mini-MAC	M±SD	Me	Q1-Q3
**Education**			
**Anxious preoccupation**			
primary – A (n=24)	20.08±3.72	20	17.75-23
vocational – B (n=48)	16.96±4.94	17.5	13.75-20
secondary – C (n=47)	16.53±4.04	17	14-19
higher – D (n=31)	15.71±3.71	15	14-19
p	p=0.002 *	-	A>B,C,D
**Fighting spirit**			
primary – A (n=24)	21.06±3.79	21	19-23.08
vocational – B (n=48)	22.23±2.62	22	21-24
secondary – C (n=47)	22.64±3.12	23	21-25
higher – D (n=31)	22.48±3.3	23	21-25
p	p=0.281	-	-
**Helplessness-hopelessness**			
primary – A (n=24)	16.92±4.07	16.5	14-21
vocational – B (n=48)	13.5±3.7	13	11-15
secondary – C (n=47)	11.98±3.72	11	9-15
higher – D (=31)	11.42±3.41	11	9-13.5
p	p<0.001 *	-	A>B,C,D B>D
**Positive re-evaluation**			
primary – A (n=24)	21.17±3.03	20.5	19.75-24
vocational – B (n=48)	21.72±2.62	22	20-24
secondary – C (n=47)	21.49±2.88	21	19-24
higher – D (n=31)	20.37±3.77	21	17.75-23
p	p=0.328	-	-
**Constructive style**			
primary – A (n=24)	42.22±5.83	42.5	39.75-45
vocational – B (n=48)	43.94±3.95	43.5	41.75-46.25
secondary – C (n=47)	44.13±5	44	40-47.5
higher – D (n=31)	42.85±6.08	44	39.5-47
p	p=0.563	-	-
**Destructive style**			
primary – A (n=24)	37±7.41	37	32-43.25
vocational – B (n=48)	30.46±7.86	30.5	26-35
secondary – C (n=47)	28.51±6.92	29	23.5-32.5
higher – D (n=31)	27.13±5.57	27	24-30
p	p<0.001 *	-	A>B,C,D B>D

**Marital status**			
**Anxious preoccupation**			
in a formal relationship – A (n=95)	16.48±4.16	17	14-19
in an informal relationship	15.89±4.14	16	13.5-18.5
single – C (n=36)	19.22±4.64	20	17-22.25
p	p=0.002 *	-	C>A,B
**Fighting spirit**			
in a formal relationship – A (n=95)	22.48±2.96	23	21-25
in an informal relationship – B (n=19)	22.47±3.19	23	21-25
single – C (n=36)	21.42±3.51	21	20-23.25
p	p=0.235	-	-
**Helplessness-hopelessness**			
in a formal relationship – A (n=95)	12.11±3.41	12	9.5-15
in an informal relationship – B (n=19)	13.47±4.14	13	11-15
single – C (n=36)	15.69±4.69	14	13-20.25
p	p<0.001 *	-	C>A
**Positive re-evaluation**			
in a formal relationship – A (n=95)	21.61±3.07	22	19.5-24
in an informal relationship – B (n=19)	21.07±3.17	21	18.5-24
single – C (N=36)	20.53±2.84	21	19-22.25
p	p=0.214	-	-
**Constructive style**			
in a formal relationship – A (n=95)	44.08±4.91	44	41-47.5
in an informal relationship – B (n=19)	43.54±4.96	44	40-47
single – C (n=36)	41.94±5.42	42	40-45
p	p=0.135	-	-
**Destructive style**			
in a formal relationship – A (n=95)	28.59±6.62	28	24-33
in an informal relationship – B (n=19)	29.37±7.28	29	25-33.5
single – C (n=36)	34.92±8.78	34	29-42
p	p<0.001 *	-	C>B,A

n – the number of respondents, p – statistical significance coefficient, M – mean, SD – standard deviation, Me – median, Q1 – lower quartile, Q2 – upper quartile

Table S4. Mental adjustment to cancer according to Mini-MAC–medical data.

**Mini-MAC**	**M±SD**	**Me**	**Q1-Q3**
**Frequency of hospitalization**			
**Anxious preoccupation**			
once - A (n=94)	16.85±4.3	17	14-20
2-5 times - B (n=38)	17.63±4.53	19	15-20
more than 5 times – C (n=17)	17.12±5.12	17	13-20
p	p=0.43	-	-
**Fighting spirit**			
once - A (n=94)	22.57±3.32	23	21-25
2-5 times - B (n=38)	22.14±2.17	22	21 - 23.25
more than 5 times – C (n=17)	20.71±3.55	21	19-23
p	p=0.092	-	-

**Helplessness-hopelessness**			
once - A (n=94)	12.4±4.04	12	9 - 15
2-5 times - B (n=38)	14.37±3.77	14	12 - 17
More than 5 times – C (n=17)	14.35±4.54	14	12 - 15
p	p=0.013*	-	B>A
**Positive re-evaluation**			
once - A (n=94)	21.69±3.04	22	20-24
2-5 times - B (n=38)	20.73±2.67	20	19-22
More than 5 times – C (n=17)	20.47±3.54	20	19-23
p	p=0.096	-	-
**Constructive style**			
once - A (n=94)	44.27±5.22	44	41-47
2-5 times - B (n=38)	42.87±3.72	42	40-45
More than 5 times – C (n=17)	41.18±6	43	36-45
p	p=0.062	-	-
**Destructive style**			
once - A (n=94)	29.26±7.63	28.5	24-34
2-5 times - B (n=38)	32±7.28	33	27.25-37-75
More than 5 times – C (n=17)	31.47±8.75	31	26-33
p	p=0.089	-	-
**Type of treatment - chemotherapy**			
**Anxious preoccupation**			
no chemotherapy (n=130)	16.78±4.27	17	14-20
chemotherapy (n=20)	18.9±5.04	18.5	15-24
p	p=0.124	-	-
**Fighting spirit**			
no chemotherapy (n=130)	22.51±3.07	23	21-25
chemotherapy (n=20)	20.35±2.98	21	18.75-22
p	p=0.005 *	-	-
**Helplessness-hopelessness**			
no chemotherapy (n=130)	12.65±3.81	13	10-15
chemotherapy (n=20)	16.3±4.64	15	12.75-21.25
p	p=0.001 *	-	-
**Positive re-evaluation**			
no chemotherapy (n=130)	21.53±3	22	19.25-24
chemotherapy (n=20)	19.68±2.88	19.5	18-21
p	p=0.006 *	-	-
**Constructive style**			
no chemotherapy (n=130)	44.04±4.9	44	41-47
chemotherapy (n=20)	40.02±4.99	40	36-43
p	p=0.001 *		-
**Destructive style**			
no chemotherapy (n=130)	29.44±7.16	29	25-34
chemotherapy (n=20)	35.2±9.31	33.5	29-46.25
p	p=0.012 *	-	-
**Type of procedure**			
**Anxious preoccupation**			
TURB – A (n=46)	15.37±4.31	15	12-18.75
cystectomy – B (n=11)	21.45±4.84	23	19-2405

nephrectomy – C (n=25)	17.6±4.18	17	15-20
NSS – D (n=17)	16.82±3.83	17	15-19
prostatectomy - E (n=49)	17.47±4.13	17	15-20
p	p=0.002 *	-	E>A<br>B>C,E,D,A
**Fighting spirit**			
TURB – A (n=46)	22.75±3.19	23	21-25
cystectomy – B (n=11)	20.36±3.17	20	18.5-21.5
nephrectomy – C (n=25)	22.44±3.03	22	21-24
NSS – D (n=17)	22.76±2.68	23	21-24
prostatectomy - E (n=49)	22.02±3	22	20-24
p	p=0.114	-	-
**Helplessness-hopelessness**			
TURB – A (n=46)	13.13±3.67	13.5	11-15
cystectomy – B (n=11)	17.73±4.92	19	13-22
nephrectomy – C (n=25)	13.6±3.79	13	11-16
NSS – D (n=17)	11.82±4.35	11	9-14
prostatectomy - E (n=49)	12.29±3.85	12	10-15
p	p=0.011 *	-	B>A,C,E,D
**Positive re-evaluation**			
TURB – A (n=46)	21.36±2.8	21	19-24
cystectomy – B (n=11)	18.86±2.45	20	18.25-20
nephrectomy – C (n=25)	21.52±3.19	22	20-23
NSS – D (n=17)	21.41±2.65	21	20-22
prostatectomy - E (n=49)	21.55±3.2	22	19-24
p	p=0.111	-	-
**Constructive style**			
TURB – A (n=46)	44.1±4.91	44	40-47.25
cystectomy – B (n=11)	39.23±3.66	40	36-42
nephrectomy – C (n=25)	43.96±4.95	43	41-46
NSS – D (n=17)	44.18±3.68	44	43-47
prostatectomy - E (n=49)	43.57±5.36	44	40-47
p	p=0.034 *	-	D,A,E,C>B
**Destructive style**			
TURB – A (n=46)	28.5±6.96	29	24-33
cystectomy – B (n=11)	39.18±9.34	42	33-4705
nephrectomy – C (n=25)	31.2±6.96	31	27-34
NSS – D (n=17)	28.65±7.62	28	24-32
prostatectomy - E (n=49)	29.76±7.32	29	26-35
p	p=0.012 *	-	B>C,E,A,D

n – the number of respondents, p – statistical significance coefficient, M – mean, SD – standard deviation, Me – median, Q1 – lower quartile, Q2 – upper quartile
